# Self-medication practice with modern and herbal medicines and associated factors among pregnant women attending antenatal care at Mizan-Tepi University Teaching Hospital, Southwest Ethiopia

**DOI:** 10.1016/j.heliyon.2022.e10398

**Published:** 2022-08-24

**Authors:** Semere Welday Kahssay, Getnet Tadege, Fewaz Muhammed

**Affiliations:** Department of Pharmaceutical Chemistry and Pharmacognosy, School of Pharmacy, College of Medicine and Health Sciences, Mizan-Tepi University, Mizan-Aman, Ethiopia

**Keywords:** Self-medication, Modern medicine, Herbal medicine, Pregnant women, ANC, MTUTH

## Abstract

**Introduction:**

Practicing self-medication using conventional and/or herbal drugs during pregnancy could contribute/result in illness and death for the mother and embryo. The focus of the current study was to investigate the level of practice and factors affecting self-medication with conventional and herbal drugs among pregnant women who were on follow-up at the antenatal care (ANC) clinic of Mizan-Tepi University teaching hospital (MTUTH), Southwest Ethiopia.

**Methodology:**

A cross-sectional study was conducted from January 1st to February 30th, 2022, among 264 pregnant mothers who were on follow-up at antenatal care of MTUTH. A lottery method was used to pick study subjects who fulfilled the inclusion criteria. An interviewer-administered structured questionnaire was used to collect data which was entered and analyzed using SPSS version 24 software. Bivariate followed by multivariate logistic regression was employed to point out factors affecting self-medication practice with conventional and herbal drugs. P-value <0.05 in multivariate analysis was used as a cut-off point to decide statistical significance.

**Results:**

It was revealed that 44.3 percent and 49.2 percent of pregnant women self-medicate with conventional and herbal medications, respectively. Women with no history of self-medication were 6.69 folds less likely to practice self-medication using conventional medicine than those with prior experience (AOR: 6.69, 95% CI, (3.847–11.659). Having no health insurance increased the odds of self-medication using conventional medicine by about 46% among pregnant women (AOR: 0.687, 95% CI, (0.373–1.264). Pregnant mothers who joined college/university education were more likely to practice self-medication than mothers without formal education (AOR: 0.656, 95% CI, (0.304–1.414). Gravidity, education level, and history of herbal medicine use were factors that influenced pregnant mothers’ use of herbal medicines.

**Conclusion:**

According to the present investigation, self-medication by expectant mothers is very common; thus, education and guidance of pregnant women and their partners should be considered during their ANC follow-up to minimize self-medication-associated potential threats.

## 1 Introduction

“Self-medication is defined as the use of drugs by patients without a medical practitioner's consent to treat self-recognized diseases or symptoms, or the prolonged or irregular use of prescribed medicines to treat chronic or recurring ailments or symptoms” [1, 2, 3]. It is also expressed as abusing substances to relieve symptoms of medical conditions, including psychological or neurological disorders such as anxiety, pain, and insominia [4].

Self-medication is a global problem and has been practiced by different age groups and populations. Pregnant women are not exceptions; they practice self-medication to manage pregnancy-associated symptoms or diseases. Special focus is given during drug therapy to pregnant mothers to avoid potential harm to the mother and the unborn baby; thus, self-medication during pregnancy could cause or contribute to maternal and fetal mortality or morbidity [5].

In developing countries like Ethiopia, with a limited number and standard of medical care, the likelihood of self-medication using conventional or herbal drugs is expected to be high. Despite the high number of pregnancy-contraindicated drugs on the market, most pregnant women do not know about these drugs and their effect on them and their unborn child [6]. Similarly, insufficient proofs are available regarding the safety and efficacy of herbal drug use in pregnant mothers [7].

*In vivo* experimental research revealed that ginger (*Zingiber officinale)*, one of the most extensively used medicinal plants, administered at a dose of 1 g/kg or higher caused subfertility and abortion in mice [8]. According to a study from Pakistan, pregnant women who want to end their pregnancies take 2 g of ginger daily [9]. Regarding other herbs, the available data are often heterogeneous and show conflicting results without a clear conclusion [10].

A recent systematic review showed that the overall self-medication practice in Ethiopia ranged from 12.8% to 77.1%, with a mean of 36.8%; and the most widely used drug classes for self-medication were painkillers/antipyretics, antibacterials, gastrointestinal, and respiratory drugs for treating pain/fever, gastrointestinal and respiratory tract diseases [12]. Besides, reports from sub-Saharan Africa revealed that pregnant women's prevalence of herbal drug use varied from 2% (Tigray, Ethiopia) to 100% (Machakos, Kenya) [13]. Peoples from the developed world are also reported to practice self-medication and ranging from 0.1% (Europe) to 100% (USA) [14].

Despite the potential threats connected with pregnant women practicing self-medication, there are limited data in Ethiopia, and no evidence is available specifically in the current study area. Thus, this study aimed to investigate the magnitude of self-medication practice using conventional and herbal drugs and the contributing factors among pregnant mothers on follow-up at ANC of MTUTH.

## 2 Methodology

### 2.1 Study area and period

The study was conducted in MTUTH, which is found in Aman Town, Southwest Ethiopia, located at a distance of 568 km from the capital city of Ethiopia, Addis Ababa. This large teaching hospital serves the people of Mizan-Aman town and the surrounding community and provides health care services for more than 2.75 million people. From the different wards and clinics available in MTUTH, this research was carried out at the ANC clinic from January 1st to February 30th, 2022.

### 2.2 Study design

Pregnant women on follow-up at ANC of MTUTH during the study period were interviewed using standardized questionnaire as part of a cross-sectional study.

### 2.3 Study population

The study included all expectant mothers who were mentally stable, granted their consent to participate, and visited the ANC of MTUTH during the study period. On the other hand, the study did not include pregnant mothers who were mentally ill or unstable.

### 2.4 Sample size determination and sampling technique

The sample size was determined using the single population proportion formula:

$$n=\frac{Z^{2}\left(1-\text{P}\right)\text{P}}{{\text{d}}^{2}}$$

where: n = sample size, p = prevalence of self-medication among pregnant women d = margin of sampling error tolerated, z = the standard normal value at a confidence interval of 95%

In a study done in 2015 among pregnant women attending antenatal care at Hosanna town, southern Ethiopia, out of 353 pregnant women included in the study, the prevalence of herbal medicine use was 73.1% [25]. This value was taken to determine the sample size.

So, n= [(1.96)^2^ (1 − 0.731)∗0.731]/(0.05)^2^ = [3.8416∗0.1966]/0.0025 = 302.

Since the total number of pregnant women on follow-up at ANC clinic of MTUTH is less than 10,000, which is 1512;

$$nf=\frac{\text{n}}{1+\frac{\text{n}}{\text{N}}}\;=nf=\frac{302}{1+\frac{302}{1512}}=251.67$$

where; nf = final sample size, n = total study population, N = total source population.

When a 5% contingency (non-response rate) is added, the final total sample size was calculated to be 264. The study subjects who fulfilled the inclusion criteria were selected by lottery method (simple random sampling technique).

### 2.5 Data collection tool and procedure

Data were collected by using an interviewer-administered structured questionnaire. The questionnaire consisted of all relevant variables, which were classified into four sections. The first section was designed to assess the socio-demographic variables of the respondents. The second, third, and fourth sections were prepared to assess obstetrics characteristics, and previous and current self-medication practices of modern and herbal medicine, respectively. Two graduating class pharmacy students collected the data with the main investigator's daily guidance.

### 2.6 Data quality control, processing, and analysis

A pretest was done in 5% of the study population to improve the questionnaire's validity and reliability and then pinpointed problems were addressed accordingly. The collected data were cleared and checked daily for consistency and completeness and then entered and analyzed using SPSS version 24. The variables' frequency and relative proportion/percentage were calculated using descriptive statistics. Bivariate followed by multivariate analysis was applied to identify factors affecting self-medication practice with modern and herbal drugs. Following bivariate analysis, predictor variables with a p-value less than 0.25 were taken into account for multivariate logistic regression analysis. In multivariate logistic regression, co-variates (predictor variables) with a p-value less than 0.05 were deemed to have a significant correlation with the outcome variables. Graphs were sketched using Graphpad prism version 8.4.3 software.

### 2.7 Definitions of terms

#### 2.7.1 Herbs

Any part of plant materials such as leaves, fruits, flowers, seeds, wood, stems, bark, rhizomes, roots, or other parts or soil materials.

#### 2.7.2 Herbal medicine

Any herb or combination of herbs used for diagnosis, curative, treatment, or prophylaxis of disease and symptoms in humans.

#### 2.7.3 Current pregnancy

Indicates mothers who were pregnant during data collection.

### 2.8 Ethical consideration

Ethical clearance was received from Mizan-Tepi University Research and Ethical Review Committee (MTU/CMHS/01357/14). Before data collection, the study's objective was explained, and written informed consent was obtained from the study subjects. Information provided by the study subjects was kept confidential, and they were also provided with the opportunity to ask questions and withdraw/decline from participation.

## 3 Results

### 3.1 Socio-demographic characteristics of the respondents

The study comprised 264 pregnant women with a complete response rate. . Of the study subjects, the majority (44.7%) were in the age group of 23–28 years with a median age of 25.5 years, and almost all (95.1%) were married. Regarding their occupation, 46.2% were homemakers, and 73.1% had poor monthly income (based on the WHO income scale level). In addition, 78 (29.5%) of them attended primary (1–8) education, 107 (40.5%) were Bench by ethnicity, and 120 (45.5%) were protestants (Table 1).

**Table 1:** Socio-demographic characteristics of pregnant women attending ANC at MTUTH, from January to February 2022 (n = 264**)**.

Variables		frequency	Percent %
Age	17–22	83	31.4
	23–28	118	44.7
	29–34	48	18.2
	≥35	15	5.7
Marital status	Married	251	95.1
	Widowed	1	0.4
	Single	4	1.5
	Divorced	8	3.0
Monthly income (ETB)∗	<1380 (poor)	193	73.1
	1381-6900 (low)	70	26.5
	6901-13800 (mid)	1	0.4
Occupation	Governmental employed	47	17.8
	Self-employed	16	6.1
	Housewife	122	46.2
	Farmer	26	9.8
	Student	53	20.1
Educational level	Illiterate	45	17
	Primary school	78	29.5
	Secondary school	69	26.1
	College/University	72	27.3
Ethnicity	Bench	107	40.5
	Amhara	102	38.6
	Kafa	33	12.5
	Others	22	8.3
Religion	Orthodox	76	28.8
	Muslim	68	25.8
	Protestant	120	45.5
Residence	Urban	196	74.2
	Rural	68	25.8
Distance	<5 km	142	53.8
	5–10 km	73	27.6
	>10 km	49	18.6
Insurance	Yes	80	30.3
	No	184	69.7

∗ classification is according to the WHO income level scale for developing countries; ETB, Ethiopian birr.

### 3.2 Obstetric information of the respondents

Among the 264 pregnant women interviewed, 97 (36.7%) were with two gravid, and 59 (22.3%) were with three and above gravid. Of a total of 196 pregnant women with two and above gravid, 87 (33%) had one child, 49 (18.6%) had three and above children, and 43 (16.3%) of them had a prior abortion history (Table 2).

**Table 2:** Obstetric characteristics of pregnant women attending ANC at MTUTH from January to February 2022 (n = 264).

Variable		Frequency	Percent (%)
Number of gravidities	One	68	25.8
	Two	97	36.7
	Three	40	15.2
	More than three	59	22.3
	Total	264	100
Number of children	No child	84	31.8
	One child	87	33
	Two child	44	16.6
	More than two	49	18.6
	Total	264	100
History of abortion	No	221	83.7
	Yes	43	16.3
	Total	264	100
Stage of pregnancy	First trimester	46	17.4
	Second trimester	120	45.5
	Third trimester	98	37.1
	Total	264	100

**Figure 1:**
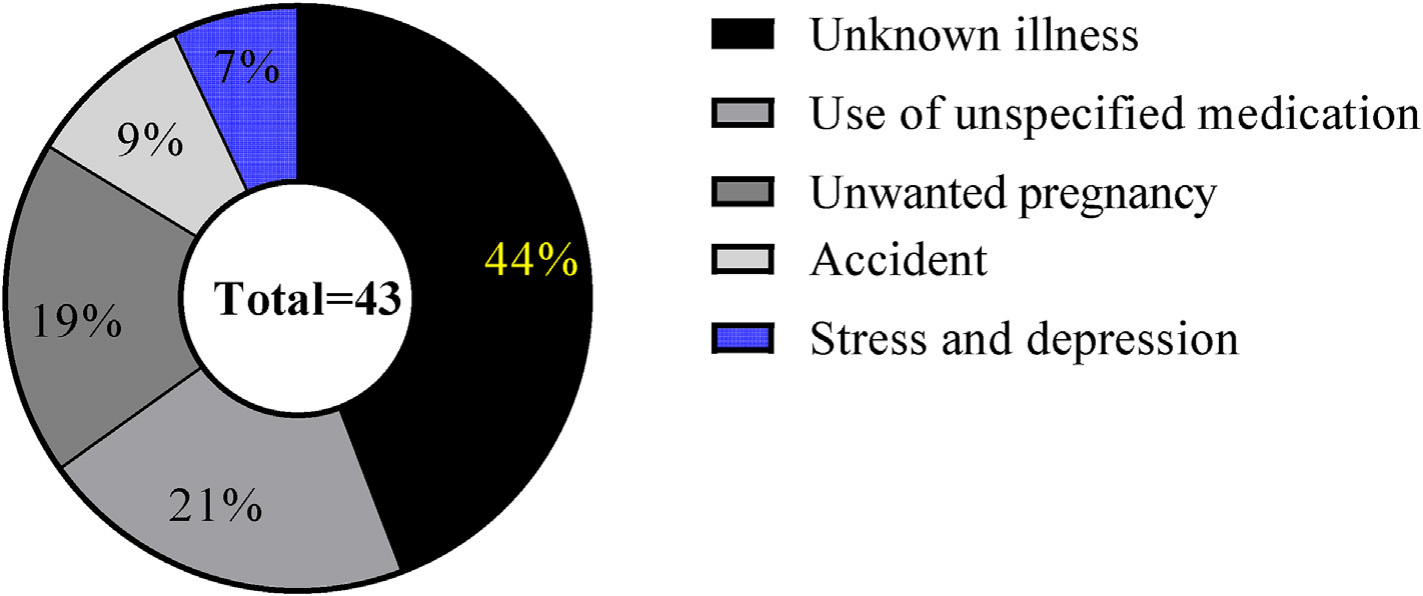
Reasons for abortion during pregnancy among pregnant women attending ANC at MTUTH, from January to February 2022 (n = 43).

As shown in Figure 1, of the total 43 pregnant women with a history of previous abortion, 19 (44.19%) of them were due to unknown illnesses, 9 (20.9%) and 8 (18.6%) of them were due to the use of unspecified medication and unwanted pregnancy, respectively.

### 3.3 Self-medication practice with modern medicines

Among the study subjects, 117 (44.3%) practiced self-medication with modern medicines during their current pregnancy. On the other hand, from a total of 196 pregnant women with multigravida, 130 (66.32%) of them exercised self-medication during their previous pregnancy. Easily availability/accessibility of drugs (44.44%) followed by saving time (19.66%) and past experience with the drug (14.53%) were the most prevalent justifications for exercising self-medication with modern medicines among expectant mothers.

**Figure 2:**
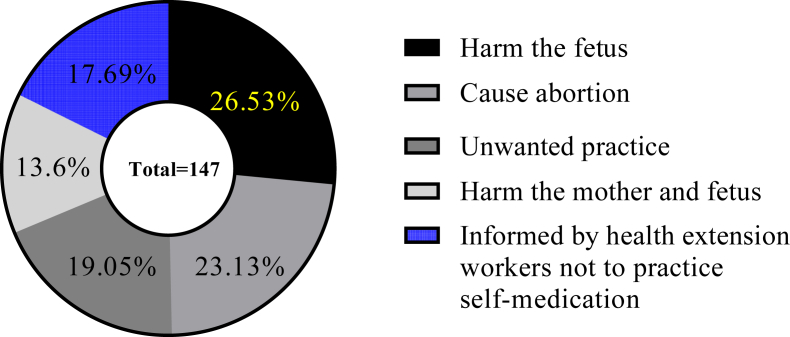
Justifications for refraining from self-medication during pregnancy among pregnant women attending ANC at MTUTH, from January to February 2022 (n = 147).

Of the participants who didn't practice self-medication during their current pregnancy (147), 39 (26.5%) and 34 (23.13%) of them believed that self-medication during pregnancy may harm the fetus and causes abortion, respectively. 26 (17.69%) of the participants didn't exercise self-medication because of the information they got from health extension workers (Figure 2).

**Figure 3:**
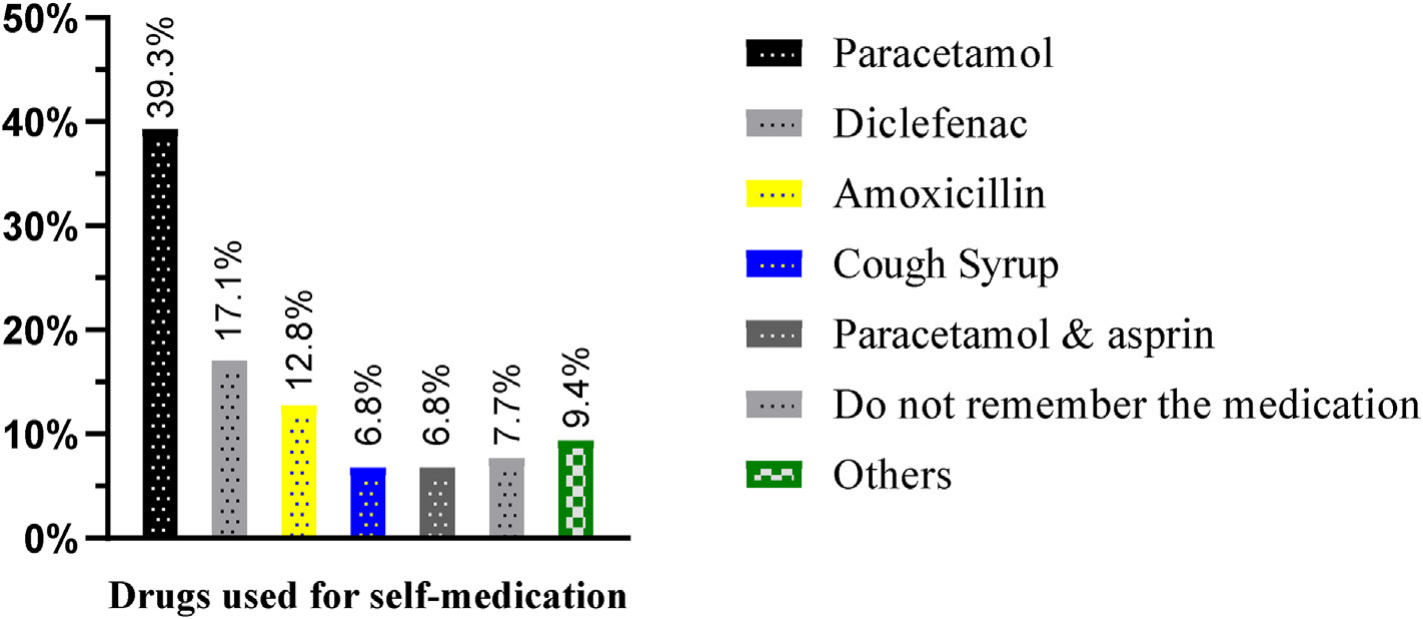
Modern medications used for self-medication among pregnant women attending ANC at MTUTH, from January to February 2022 (n = 117).

Of the 117 pregnant women who exercised self-medication during their current pregnancy, 52.14% used the drug to treat headache, followed by common cold (14.53%) (Table 3). As depicted in Figure 3, paracetamol (39.32%), diclofenac (17.1%), and amoxicillin (12.8%) were the three most commonly utilized medications for self-medication by pregnant women. A small proportion of the participants (7.7%) didn't remember the type of medication they used.

**Table 3:** Indication for practicing self-medication among pregnant women attending ANC at MTUTH, from January to February 2022, (n = 117).

Indication for self-medication	Frequency	Percent
Headache	61	52.14
Common cold	17	14.53
Headache and cough	13	11.1
Cough and diarrhea	10	8.55
Others^@^	16	13.67
Total	117	100

@thyphoid, UTI, diarrhea, headache + common cold, NV + common cold, H + N.

**Figure 4:**
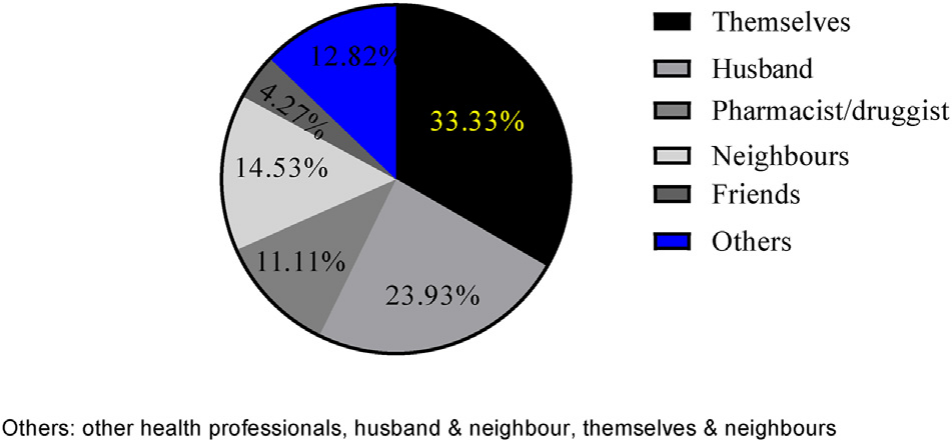
Sources of information for self-medication with modern drugs among pregnant women attending ANC at MTUTH, from January to February 2022, (n = 117).

**Figure 5:**
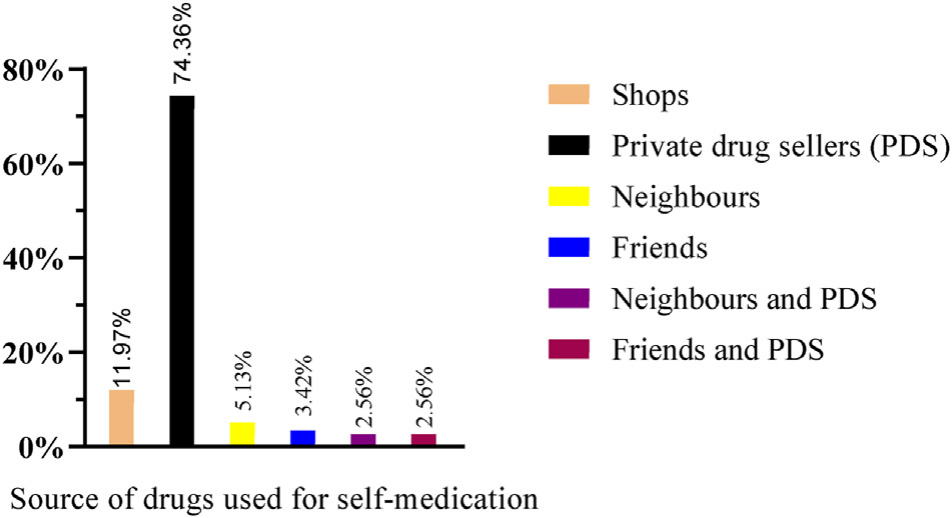
Sources of modern drugs for self-medication among pregnant women attending ANC at MTUTH, from January to February 2022, (n = 117).

Among the pregnant women with a history of self-medication during their current pregnancy, 33.33% and 23.93% of them stated that the primary source of knowledge for self-medication was themselves and their husbands, respectively (Figure 4). A large proportion of the participants (74.36%) obtained the drugs from community drug retail outlets (CDROs), while 11.96% got them from shops (Figure 5). Badly, more than half of these pregnant mothers, 69 (58.97%), had no information about the drugs they were taking, while 29 (24.79%) and 18 (15.38%) of them knew how to take and dose of the drug, respectively.

### 3.4 Factors associated with self-medication practice using modern medicine.

In the present study, multivariate logistic regression analysis revealed a strong relationship between current self-medication practices utilizing modern medicine and education level, insurance, and prior history of self-medication. Women with no history of self-medication were 6.69 folds less likely to practice self-medication with modern medicine than their counterparts (AOR: 6.69, 95% CI, (3.847–11.659). The study also revealed that having no health insurance increased the odds of self-medication with modern medicine by about 46% among pregnant women (AOR: 0.687, 95% CI, (0.373–1.264). Unlike women who attended primary and secondary school, pregnant mothers who joined college/university education were more likely to practice self-medication than mothers without formal education (AOR: 0.656, 95% CI, (0.304–1.414) (Table 4).

**Table 4:** Factors associated with self-medication practice with modern medicine during pregnancy among pregnant women attending ANC at MTUTH, from January to February 2022.

	Variable	Self-medication		Binary logistic regression analysis (COR, 95% CI)	Multivariate L.regression analysis (AOR, 95% CI)
		Yes	No		
Occupation	Gov. employed	18 (15.4%)	29 (19.7%)	1.0	1.0
	Self-employed	8 (6.8%)	8 (5.4%)	0.621 (0.198–1.946)	0.375 (0.13–1.08)
	Housewife	58 (49.6%)	64 (43.5%)	0.685 (0.345–1.362)	0.891 (0.24–3.27)
	Farmer	14 (12.0%)	12 (8.2%)	0.532 (0.202–1.403)	0.795 (0.324–1.95)
	Student	19 (16.2%)	34 (23.2%)	1.1 (0.493–2.5)	0.632 (0.16–2.48)
Education	Illiterate	26 (22.2%)	19 (12.9%)	1.0	1.0
	Primary school	39 (33.3%)	39 (26.5%)	1.368 (0.653–2.867)	1.713 (0.737–3.98)∗
	Secondary school	22 (18.8%)	47 (32.0%)	2.923 (1.34–6.369)	1.313 (0.636–2.709)∗
	College/university	30 (25.6%)	42 (28.6%)	1.916 (0.9–4.07)	0.656 (0.304–1.414)∗
Insurance	Yes	29 (24.8%)	51 (34.7%)	1.0	1.0
	No	88 (75.2%)	96 (65.3%)	0.62 (0.362–1.064)	0.687 (0.373–1.264)∗
Stage of pregnancy	First trimester	22 (18.8%)	24 (16.3%)	1.0	1.0
	Second trimester	56 (47.9%)	64 (43.6%)	1.048 (0.53–2.069)	0.892 (0.387–2.055)
	Third trimester	39 (33.3%)	59 (40.1%)	1.387 (0.685–2.809)	0.986 (0.527–1.845)
Prior self-medication	Yes	87 (74.4%)	43 (29.3%)	1.0	1.0
	No	30 (25.6%)	104 (70.7%)	7 (4.06–12.1)	6.69 (3.847–11.659)∗

The predicted probability is for not practicing self-medication with modern medicine, ∗ indicates statistical significance at p ≤ 0.05.

### 3.5 Self-medication practice using herbal medicines

Almost half of all the respondents, 130 (49.2%), used herbal medicine during their current pregnancy. 95 (48.5%) of the 196 pregnant women with multigravida had previously used herbal medications.. Among the 134 pregnant women who did not utilize herbal medicine during their current pregnancy, the majority (41.79%) believed that herbal medicine is unwanted practice, and 17.9% of them suspected that the use of herbal medicine couldcause harm to their fetus (Table 5).

**Table 5:** Reason for not practicing herbal medicine among pregnant women attending ANC at MTUTH, from January to February 2022, (n = 134).

Reason for not using herbal medicine	Frequency(N)	Percentage (%)
Undesirable practice	56	41.79
Possibility of harm to the fetus	24	17.9
Have no knowledge of using herbal remedies	19	14.2
Might cause abortion	18	13.43
Harm the mother and her fetus	8	5.97
Difficult to determine the dosage of herbal medicine	7	5.22
May harm to the fetus and have no knowledge about herbal medicine use	1	0.75
May be harmful to the fetus and cause abortion	1	0.75
Total	134	100

**Figure 6:**
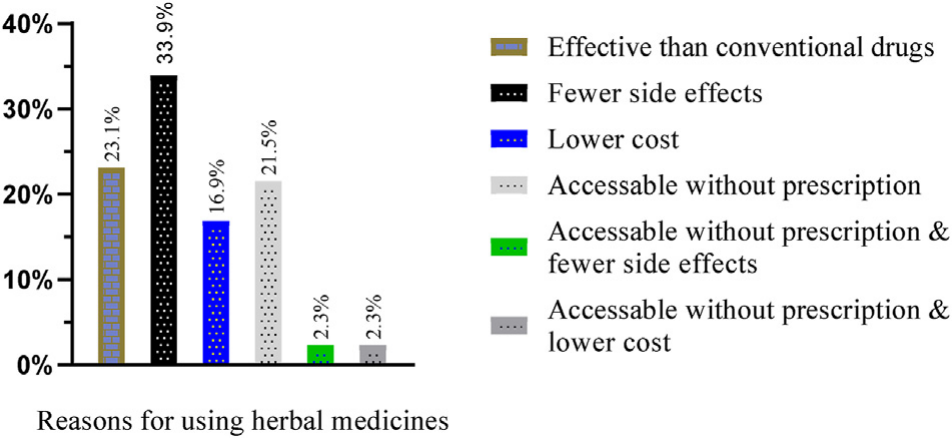
Reasons for using herbal medicine among pregnant women attending ANC at MTUTH, from January to February 2022, (n = 130).

**Figure 7:**
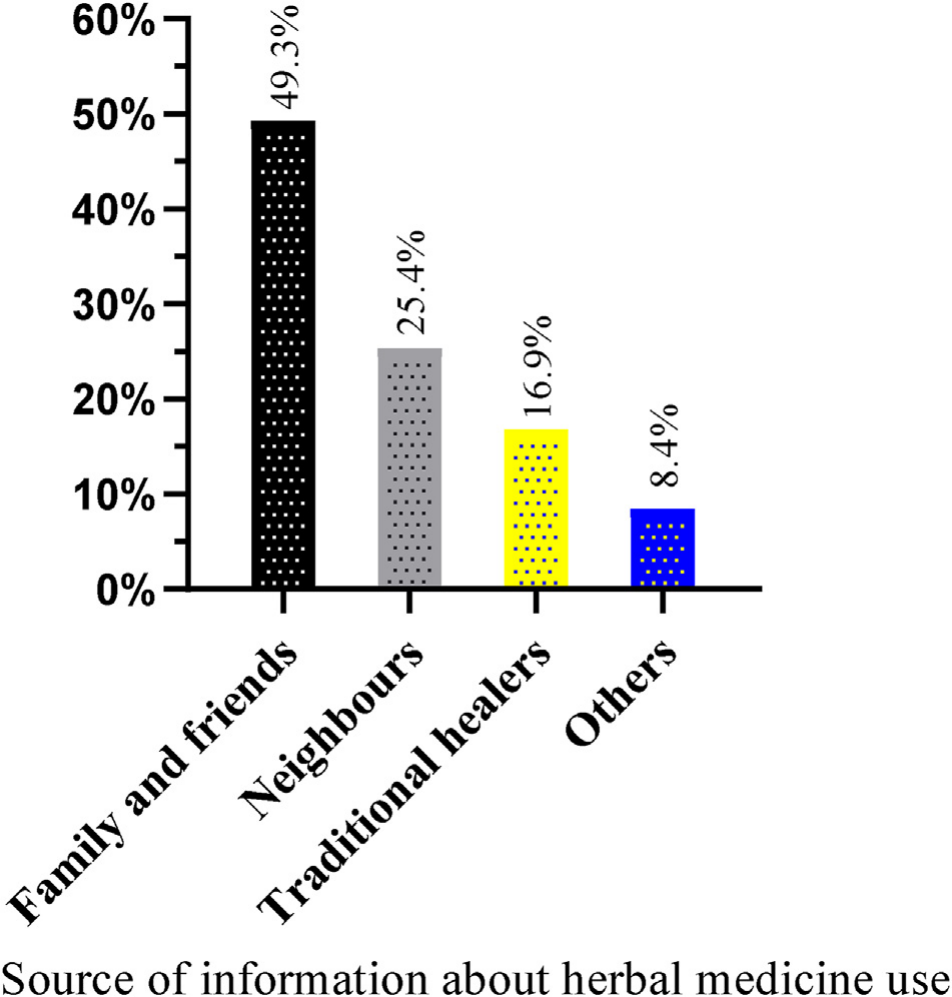
Source of information about herbal medicine use among pregnant women attending ANC at MTUTH, from January to February 2022, (n = 130).

**Figure 8:**
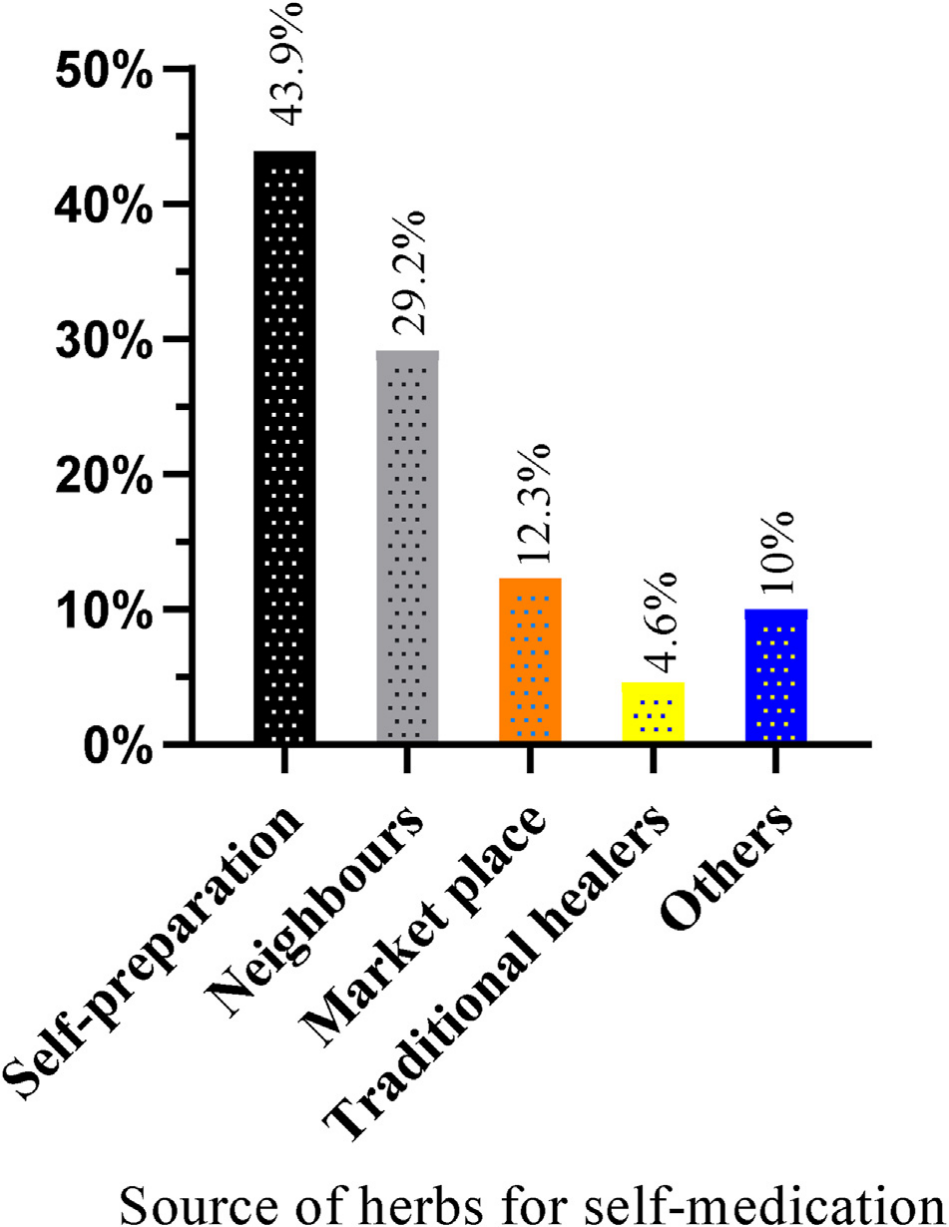
Source of herbs for self-medication among pregnant women attending ANC at MTUTH, from January to February 2022, (n = 130).

44 (33.85%) of the pregnant women who used herbal medicine during their most recent pregnancy believed that herbal medicines have fewer adverse effects. . In comparison, 23.08% of them perceived that herbal medicines are more beneficial than prescription drugs (Figure 6). Most of the women, 30.0%, used the herbs to treat nausea and vomiting, followed by common cold (26.92%). The most widely used herb was dama-kesse (*Ocimum Lamiifolium*) (22.31%), followed by ginger (*Zingiber officinale*) (17.69%) and garlic (*Allium sativum*) (16.15%) (Table 6). Almost half of these study subjects, 64 (49.23%), obtained information on herbal medicines from their family and friends. Most of the pregnant mothers, 57 (43.85%), used self-prepared herbal medicines. On the contrary, a relatively small proportion of these study subjects, 16.9%, and 4.6%, used traditional healers as a source of information about herbal medicine use and as a source of herbs, respectively (Figures 7 and 8). The type of herbs with their reported benefits and preparation are shown in Table 7.

**Table 6:** Indication for herbal medicine use and name of herbs used among pregnant women attending ANC at MTUTH, from January to February 2022, (n = 130).

Herbal medicine		Frequency	Percent (%)
**Indications**	Headache	13	10
	Nausea and vomiting	39	30
	Common cold	35	26.92
	To prevent abortion	7	5.38
	Combination of two ©	17	13.07
	Others ^♀^	19	14.62
	**Total**	**130**	100
**Name of herbs used**	Ginger (*Zingiber officinale*)	23	17.69
	Garlic (*Allium sativum*)	21	16.15
	Dama-kesse (*Ocimum Lamiifolium*)	29	22.31
	Tena Adam *(Ruta chalepenesis)*	10	7.69
	Tosign (*Thymus schimperi*)	8	6.15
	Ginger and garlic	17	13.07
	Ginger and Tena Adam	10	7.69
	Others^☼^	12	9.23
	**Total**	**130**	100

©: common cold + prevent abortion, NV + CC, prevent abortion + NV, H + CC, …

♀: allergic, diarrhea, typhoid…

☼: garlic and tena-adam, dama-kesse and tosign, ginger and dama-kesse .

**Table 7:** Reported uses and preparation of herbal medicines by pregnant mothers attending ANC at MTUTH, from January to February 2022, (n = 130).

Type of herb	Reported uses by pregnant mothers	Preparation
Ginger (*Zingiber officinale*)	Morning sickness, cough during common old, aid digestion	Steeping sliced or grated fresh ginger in hot water (ginger tea)
Garlic (*Allium sativum*)	Common cold, flu, prevention of preeclampsia	Adding it to a dish
Dama-kesse (*Ocimum Lamiifolium*)	Headache, fever, hypertension, and flank pain.	Sniffing or Squeezing the leaf and adding it into tea/coffee
Tena Adam *(Ruta chalepenesis)*	Headache, fever, and cold	Adding the fresh leaf into tea/coffee
Tosign (*Thymus schimperi*)	Cough, stomach pain, and as a flavoring agent	Fresh herbs are boiled in tea

### 3.6 Factors contributing to self-medication practice with herbal medicines

Multivariate logistic regression analysis revealed that gravidity, prior history of herbal medicine use, and education level were factors that affected pregnant mother's use of herbal medicines. The odds of self-medication with herbal medicine decreased by about 95% among pregnant mothers with two gravid than women with primigravida (one gravid) (AOR: 23.14, 95% CI, (0.94–568.9)). The analysis also showed that the odds of herbal medicine use was very low among pregnant women with no history of herbal medicine use (AOR: 26.95, 95% CI, (10.75–67.52)). The finding revealed that, among literate mothers, those who attended college/university were more likely to practice self-medication with herbal medicines than mothers without formal education (Table 8).

**Table 8:** Factors associated with herbal medicine use among pregnant women attending ANC at MTUTH, from January to February 2022.

	Variables	Herbal medicine use		Bivariate analysis (COR, 95% CI)	Multivariate analysis (AOR, 95% CI)
		Yes	No		
Education	Illiterate	34 (26.2%)	11 (8.2%)	1.0	1.0
	Primary school	38 (29.2%)	40 (29.9%)	3.25 (1.45–7.33)	3.99 (1.18–13.44∗
	Secondary school	28 (21.5%)	41 (30.6%)	4.97 (1.969–10.4)	1.33 (0.6–2.94)∗
	College/University	30 (23.1%)	42 (31.3%)	4.327 (1.89–9.88)	0.83 (0.38–1.80)∗
Place of residence	Urban	87 (66.9%)	109 (81.3%)	1.0	1.0
	Rural	43 (33.1%)	25 (18.7%)	0.46 (0.263–0.82)	0.99 (0.45–2.17)
Gravid	One gravid	20.8%	30.6%	1.0	1.0
	Two gravid	33.8%	39.6%	0.79 (0.42–1.48)	23.15 (0.94–568.9)∗
	Three gravid	18.5%	11.9%	0.44 (0.98–0.97)	3.47 (0.22–54.78)∗
	>3 gravid	26.9%	17.9%	0.452 (0.22–0.92)	5.15 (0.57–46.18)∗
Child	No child	26.1%	37..3%	1.0	1.0
	One child	31.5%	34.3%	0.76 (0.42–1.39)	0.34 (0.02–7.49)
	Two child	17.7%	15.7%	0.62 (0.298–1.3)	0.29 (0.02–4.6)
	>2 child	24.6%	12.7%	0.36 (0.174–0.75)	0.36 (0.045–2.87)
Prior herbal medicine	Yes	73.8%	23.1%	1.0	1.0
	No	26.2%	76.9%	9.38 (5.36–16.4)	26.95 (10.75–67.52)∗

The predicted probability is for not practicing self-medication with herbal medicine, ∗ indicates statistical significance at p ≤ 0.05.

## 5 Discussion

Numerous physiological changes that occur during pregnancy can cause conditions that call for medical attention. To manage these symptoms or illnesses, pregnant women practice self-medication. The goal of the current study was to determine the frequency of self-medication with conventional and herbal medications, as well as associated factors, among pregnant women visiting prenatal clinics in MTUTH.

In the present study, the prevalence of self-medication with modern/conventional medicines during current and prior pregnancies were 44.32% and 66.32%, respectively. This finding is higher than the findings at Jimma University Specialized Hospital (JUSH), Ethiopia (20.1%, 63.7%) [15], Goba town (15.5%) [16], and Gedeo Zone (40.4%) [17] but lower than the results of studies done at selected hospitals in Jos, Nigeria [6]; and health centers in Bukavu, Eastern DR Congo [5] which reported a current prevalence of 85% and 61.3%, respectively. Similar to the current study, a survey from Tanzania reported a prevalence of 46.24% [18]. These differences in the prevalence of self-medication with modern medicines during pregnancy can be attributed to differences in the socio-demographic characteristics of the participants, a country's policy in regulating prescription drugs, beliefs and culture of the society, and accessibility of health care centers and professionals.

The major justifications for using conventional drugs for self-medication were due to the easy availability of drugs (44.44%) and saving time (19.66%). The same reasons were given by the respondents in JUSH, Ethiopia [15]; health centers in Bahir Dar, Ethiopia [19]; and at hospitals in Jos, Nigeria [6]. This might be due to the high workload and low health care-seeking behaviors of women in Ethiopia [20,21].

The most prevalent symptoms or illnesses that pregnant women in this study self-medicated were headache (52.14%), common cold (14.53%), and cough (11.1%). This result was consistent with studies conducted at Kemisie [22], Jimma [15], Harar [23], DR Congo [5], and Nigeria [6], in which headache/fever, common cold, respiratory infections, and gastrointestinal disorders were among the frequent conditions for which expectant mothers used conventional drugs for self-medication.

In the current study, paracetamol (39%), diclofenac (17.01%), and amoxicillin (12.82%) were the most commonly used medications during pregnancies, and similar findings were published in Goba town [16], Gedeo Zone [17], Jimma [24], and DR Congo [5]. Over-the-counter availability of these drugs, except for amoxicillin, might be responsible for their high utilization by pregnant women.

This study pointed out that the major sources of modern drugs for self-medication were private drug retail outlets (74.36%) which is in line with the studies done at Jimma [15] and Kemisie [22], which revealed that the majority of the pregnant mothers, 72.1% and 50% obtained the medications from private drug retail outlets, respectively. Strong/strict regulation of private drug retail outlets is thus essential to secure proper utilization of medications.

Our study revealed that women with no history of self-medication were 6.69 folds less likely to practice self-medication with modern medicine than their counterparts (AOR: 6.69, 95% CI, (3.847–11.659). This finding is in parallel with the reports from Hossana [25], Harar [23], Gedeo zone [17], and Kemisie [22]. The current study also revealed that having no health insurance increased the odds of self-medication with modern medicine by about 46% among pregnant women (AOR: 0.687, 95% CI, (0.373–1.264). This result is supported by the report of Azam Rahman et al. [26] and Abdi Befikadu et al. [15], who revealed the absence of or inadequate healthcare insurance coverage promotes self-medication. This could be due to an increase in the cost of healthcare services. Unlike women who attended primary and secondary school, pregnant mothers who joined college/university education were more likely to practice self-medication than mothers with no formal education (AOR: 0.656, 95% CI, (0.304–1.414). This finding is in parallel to the reports from central Mexico [27] and France [28].

Regarding self-medication using herbal medicine**,** in this study, Pregnant women took herbal remedies at rates of 49.2% and 48.5% during their most recent and previous pregnancies, respectively. This finding is lower than a report from Harar, which reported a prevalence of 58.2% and 63.2%, respectively [23]. On the other hand, our finding is in line with the surveys done in Kemisie, Northeast Ethiopia (49.8%)^22^; Nekemte, Western Ethiopia (50.4%) [29]; and Kelantan, Malaysia (51.4%) [30]. However, it was higher than the finding of the study done in Kenya (12%) [31] and Saudi Arabia (33%) [32] but lower than the reports from Hosanna, Ethiopia (73.1%)^25^; and Ibadan, Nigeria (63.8%) [33]. This variability may be due to differences in availability, affordability, religious, and cultural issues regarding herbal and conventional medicines in different states and globally.

In the present study, the typical justifications for avoiding herbal remedies during pregnancy were considering it an unwanted practice (41.79%), fear of harm to the fetus (17.9%), and lack of knowledge on the usage of herbal remedies (14.2%), which showed variation with the study done in Harar [23] which mentioned fear of harm to the fetus (40.2%) as a major reason followed by unwanted practice (22.5%). Besides, this study disclosed that perceived fewer side effects (33.85%), more effectiveness (23.08%), and accessibility without prescription (21.54%) of herbal medicines were the common reasons stated by the pregnant mothers for self-medication using herbal medicine. This finding was in line with the study done among pregnant mothers attending ANC at Nekemte hospital [29] and Hossana Town [25].

According to this study, the most frequent causes for using herbal remedies during pregnancy were nausea/vomiting, followed by common cold and headache. . This result was similar with the research done at Nekemte Hospital [29] and Hosanna town [25]. This parallelism could be due to the similarities in the symptoms/ailments pregnant women complain during their pregnancy.

This study disclosed that dama-kesse (*Ocimum Lamiifolium*) (22.31%), ginger (*Zingiber officinale*) (17.69%), and garlic (*Allium sativum*) (16.31%) were the most common herbal medicines used during pregnancy. Similarly, studies done in Hosanna [25] and Nigeria [34] reported that garlic, ginger, tena-adam, dama-kesse, and eucalyptus were the most popular herbal remedies utilized during pregnancy. Our study subjects revealed that they had been using ginger tea to treat morning sickness, cough, and to aid digestion; sniffing or drinking the exudate of dama-kesse to treat headache, fever, flank pain, and hypertension; and garlic for common cold, flu, and prevention of preeclampsia. Their uses are supported by traditional claims of the plants as well as *in vivo* and *in vitro* experimental studies [10,13,[35], [36], [37]].

In the current study, the odds of self-medication using herbal medicine decreased with the absence of prior experience, which is in line with the researches from Bangladish [38], Gondar [39], Kemisie [22], and Bahir Dar [19]; an increase in the number of gravidities, which is consistent with the report from Bahir Dar [19]; and increase in education level, which is similar to the findings from Turkey [40] and Australia [41].

### 5.1 Limitation of the study

The cross-sectional nature of the study design limits the establishment of a cause and effect relationship between the predictor and outcome variables. Since data were collected by interviewing the study subjects, there is a potential for recall bias. These should be considered while interpreting the findings of the current study.

## 6 Conclusion

Self-medication during the current pregnancy was shown to be common in both conventional and herbal medicine, at 44.3 percent and 49.2 percent, respectively. The two most frequently cited benefits of using self-medication with modern medications were accessibility and time savings, whereas the two most popular benefits of using herbal medications among pregnant women were perceived lower side effects and higher effectiveness. Paracetamol, diclofenac, and amoxicillin were the most common drugs used for self-medication. At the same time, dama-kesse (*Ocimum Lamiifolium*), garlic (*Allium sativum*) and ginger (*Zingiber officinale*) were the most common herbs used by pregnant mothers. The use of modern medicine for self-medication was substantially correlated with education level, insurance, and prior self-medication (P < 0.05). Moreover, it was discovered that education level, gravidity, and prior use of herbal remedies all had an impact on the pregnant women's usage of herbal remedies for self-medication (P < 0.05). Generally speaking, during their ANC follow-up, pregnant women should be educated and counseled against the dangers of self-medicating with contemporary and/or herbal medications.

## Declarations

### Author contribution statement

Semere Welday Kahssay and Fewaz Muhammed Ali: Conceived and designed the experiments; Performed the experiments; Analyzed and interpreted the data; Contributed reagents, materials, analysis tools or data; Wrote the paper.

Getnet Tadege: Performed the experiments; Analyzed and interpreted the data; Contributed reagents, materials, analysis tools or data; Wrote the paper.

### Funding statement

This research did not receive any specific grant from funding agencies in the public, commercial, or not-for-profit sectors.

### Data availability statement

Data will be made available on request.

### Declaration of interest’s statement

The authors declare no conflict of interest.

### Additional information

No additional information is available for this paper.
