# Supplementary material for: Self-medication practice with modern and herbal medicines and associated factors among pregnant women attending antenatal care at Mizan-Tepi University Teaching Hospital, Southwest Ethiopia
Source: Heliyon. 2022 Aug 24;8(9):e10398. doi: 10.1016/j.heliyon.2022.e10398 (PMC9450074; doi:10.1016/j.heliyon.2022.e10398)
Supplement: Supplementary file [file mmc1.docx]

**QUESTIONNAIRE**

MIZAN-TEPI UNIVERSITY

COLLEGE OF MEDICINE AND HEALTH SCIENCES

SCHOOL OF PHARMACY

**Self-medication practice with modern and herbal medicines and associated factors among pregnant women attending antenatal care at Mizan-Tepi University Teaching Hospital, Southwest Ethiopia**

Introduction: your honest and genuine answers to these questions will have a great value to the research outcome. Your answers are completely confidential. Your name will not be written and will never be used in connection with any of the information you provide. We would greatly appreciate your help in responding to this questionnaire

**Section A: Socio-Demographic Information**

1. Age (years) _______________

2. Marital status

a) single b) married c) divorced d) widowed

3. Occupation

a) Governmental employed

b) self-employee

c) Housewife

d) Farmer f) Other ____

e) student

4. Monthly income (in ETB)_________________________________________

5. Education level

a) Illiterate

c) Secondary school (9-12)

b) Primary school (1-8)

d) College/University student

e) Diploma/Degree

6. Ethnicity

a) Bench b) Amhara c) Tigre d) Oromo e) Others _________________

7. Religion

a) Orthodox b) Muslim c) Protestant d) Others ______________

8. Place of Residence

a) Urban b) Rural

9. Distance from health facility (hospital or health center)

a) < 5km b) 5-10km c) >10km

10) do you have health insurance?

A) yes B) no

**Section B: Obstetrics Information**

1. Number of gravid ____________________________

2. Number of child _____________________________

3. Previous abortion

a) No b) Yes

4. If yes, the reason/s for abortion (specify it) ____________________

5. Stage of pregnancy

a) First trimester b) Second trimester c) Third trimester

**Section C: Self-Medication Practice using modern medicine**

1. Have you practiced self-medication using modern medicine during prior pregnancies?

a) Yes b) No

2. Have you practiced self-medication using modern medicine during the current pregnancy?

a) Yes b) No

3. If your answer in Q2 is no what is your reason ___________________________

4. What makes you to practice self-medication?

a) Time saving

b) Easily available

d) Had prior experience to the drug

e) Other reason (specify)________________

c) Better knowledge about the disease and treatment

5. For what types of ailments you have practiced self-medication using modern medicine?

a) Headache b) Nausea/Vomiting c) Typhoid d) UTI e) Common cold f) Diarrhea

g) Cough h) Other (specify)_______________________________

6. What is the name of the drug(s) you have used for self-medication?

a) Paracetamol b) Aspirin c) Tetracycline d) Amoxicillin

e) Cough syrup f) Hyoscine

g) I don’t remember h) Other (specify) ___________________

7. Who is your source of information about the drugs?

a) Yourself b) Your husband c) Your friend d) Your neighbor e) internet

f) Pharmacist/Drug gist g) other health professional h) Other (specify)__________

8. From where did you get the drugs used for self-medication?

a) Neighbors b) Friends c) Shops d) Private drug sellers (Community pharmacy and drug stores) e) Other (specify) _____________________

9. What did you know about the drugs that you have used for self-medication?

a) Dose b) Side effects c) How to take d) No information

**Section D: Herbal Medicine Use Practice**

1. Have you practiced herbal medicine during prior pregnancies?

a) Yes b) No

2. Have you practiced herbal medicine during the current pregnancy?

a) Yes b) No

3. If your answer in Q2 is no, what is your reason ___________________________

4. What makes you to practice herbal medicine use (Reason for selecting herbal medicine use)?

a) Herbal medicines are effective than conventional medicines

b) Herbal medicines have fewer side effects

c) Herbal medicines have lower cost

d) Herbal medicines are accessible without prescription

e) Other (specify)_______________________________

5. For what purpose and ailments did you used herbal medicine?

a) Headache b) Nausea/Vomiting c) Typhoid d) UTI e) Common cold f) Diarrhea

h) To prevent abortion i) Other (specify)_____________________________

6. What types of herb(s) have you used?

a) Ginger (*Zingiber officinale*)

b) Garlic (*Allium sativum*)

c) Tena-adam *(Ruta chalepenesis)*

d) Dama-kesse (*Ocimum Lamiifolium*)

e) Tosign (*Thymus schimperi*)

f) Other (specify)_________________

7. Who was your source of information about herbal medicine?

a) Traditional healers

b) Health professionals

c) Religious leaders

d) Family and friends

e) Neighbors

f) Other (specify)________________

8. From where did you get the herbal medicine?

a) Self-preparation

b) Traditional healers/Herbalist

c) Traditional birth attendants

d) Market place

e) Neighbors

f) Other (specify) ______________
